# Survival disadvantage of male children with retinoblastoma in the United States: Surveillance Epidemiology and End Results (2000–2017) Evidence

**DOI:** 10.1002/cam4.3967

**Published:** 2023-01-31

**Authors:** Laurens Holmes, Emily Pollack, Betyna N. Berice, Daniel R. Halloran, Kadedrah Parson, Nastocia T. Badfford, Lavisha Paleaz, Jacqueline A. Benson

**Affiliations:** ^1^ Nemours/Alfred I. DuPont Hospital for Children Office of Health Equity & Inclusion Health Disparities Science Research Wilmington DE USA; ^2^ Biological Science Department University of Delaware Newark NJ USA; ^3^ Thomas Jefferson University School of Population Health and Medical School Philadelphia PA USA; ^4^ Master of Public Health Dr. Kiran C. Patel College of Osteopathic Medicine Nova Southeastern University Davie FL USA; ^5^ Perelman School of Medicine University of Pennsylvania Master of Public Health Program Philadelphia PA USA

**Keywords:** epidemiology and prevention, epigenetics, health disparity pediatric cancer, retinoblastoma, SEER

## Abstract

**Background:**

Retinoblastoma is a rare malignancy involving the retina, although, more common among children, with genetic inheritance explaining the incidence as well as acquired forms. The incidence varies among race and sex as well as mortality and survival. The current study aimed to assess retinoblastoma cumulative incidence (CMI), mortality, and survival by sex.

**Methods:**

A retrospective cohort design was used to assess the CMI, mortality, and survival in this pediatric malignancy based on the Surveillance Epidemiology and End Results (SEER) data 2000–2017. The binomial regression model was used to examine sex differentials in mortality, as well as other study variables, while Cox proportional hazard model was used for the survival variability by sex.

**Results:**

The CMI during this period was higher among males relative to females (males *n* = 249, 56.7%; females *n* = 190, 43.3%, *χ* ^2^ = 2.90, *df* = 1, *p* = 0.089). There were sex differences in mortality, with excess mortality observed among males compared to females, risk ratio = 3.40, 95% CI [1.0–15.72]. The survival differences by sex indicated decreased survival among males relative to females, hazard ratio (HR) = 3.39, 95% CI [1.0–15.72]. After controlling for the potential confoundings, namely tumor grade, urbanity, and median income the survival disadvantage of males persisted. Compared to females’, males were more than three times as likely to die, adjusted HR = 3.42, 99% CI [0.37–31.60].

**Conclusion:**

In a representative sample of pediatric retinoblastoma, there was a sex differential in survival with excess risk of dying identified among males relative to females, which may be explained in part by male X‐linkage.

## Graphical abstract

Retinoblastoma is a rare malignancy involving the retina, although more common among children, with genetic inheritance explaining the incidence as well as acquired forms. The incidence varies among race and sex as well as mortality and survival. The current study aimed to assess retinoblastoma cumulative incidence, mortality, and survival by sex. In a representative sample of pediatric retinoblastoma, there was a sex differential in survival with excess risk of dying identified among males relative to females, which may be explained in X‐linkage in males.

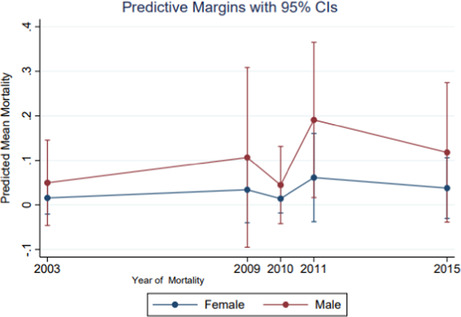

## 1 INTRODUCTION

Retinoblastoma is a rare pediatric malignancy with an estimated 4% of all malignant neoplasm among children in the United States.^1^ This malignancy involves the retinoblastoma cells due to sporadic and hereditary conditions.^1^, ^2^ Like other malignancies, retinoblastoma is primarily driven by mutation of the DNA as a result of pro‐oncogene being transformed to oncogenes and the inactivation of the tumor suppressor gene.^1^ Retinoblastoma predominantly affects children under 5 years of age, with estimated proportionate morbidity of 6%.^1^ While retinoblastoma is not an aggressive malignancy, if untreated, this malignant neoplasm results in blindness and mortality following metastasis.^1^ Although, most cases are diagnosed within the first 2 years of life, the diagnosis of unilateral disease takes on average 10–12 months longer than bilateral disease.^2^, ^3^ Epidemiological and clinical data implicate malignant retinoblastoma with both alleles’ mutation of the retinoblastoma gene. An estimated 45% of retinoblastoma involves inherited mutation in the RB1 gene.^3^, ^4^

Pediatric retinoblastoma heavily burdens low‐ and middle‐income countries.^3^ This population has fewer resources and less access to specialized care. This results in poorer prognosis and greater risk of mortality.^3^ Global health research implicates late diagnosis and limited access to care in the poor prognosis of this malignant neoplasm.^3^, ^4^ Other contributing factors to increased mortality in retinoblastoma include physician knowledge of care, availability and cost of treatment, and patient acceptance of enucleation.^4^

While there is evidence that race‐related income and health care inequities contribute to cancer disparities, there is insufficient evidence regarding pediatric retinoblastoma disparities in the United States.^5^ Specifically, there are no sufficient data on the exposure function of socioeconomic, race, and ethnicity in retinoblastoma predisposition as well as mortality.^5^, ^6^ However, most literature on pediatric retinoblastoma disparities focuses on the prevalence and management of the disease exclusively in global health efforts.^4^, ^5^ Unlike adult malignancies, environmental causes of childhood cancer are difficult to identify and complex to determine what children might have been exposed to carcinogens early in their development.^7^ If retinoblastoma is diagnosed early, this early diagnosis implies good prognosis, resulting in survival advantage relative to late stage at diagnosis and metastasis. The accurate diagnosis of retinoblastoma requires genetic testing, laboratory data, histology, physical examinations, and accurate documentation of both the family and medical history of the child.^1^, ^5^, ^6^, ^7^ Despite the implication of early diagnosis and improved prognosis, disparities exist in the management and outcomes of children with retinoblastoma, resulting in survival disadvantage for some subpopulations of children with this malignancy.^7^, ^8^ Based on current epidemiologic and clinical research data, there are no studies on sex implication as a biological variable in pediatric retinoblastoma mortality and survival.^3^

With the limited data on sex and race involvement as exposure function of pediatric retinoblastoma mortality and survival, the current study was proposed to assess these insufficiencies. Specifically, we sought to examine the cumulative incidence (CMI), mortality, and survival of children with retinoblastoma, and followed for the disease between 2000 and 2017. We postulated that there are sex differences in retinoblastoma CMI, trends, mortality and survival, and that sex disparities in these outcomes are explained in part by age, tumor prognostic factors, geography, and social determinants of health, as well as male X‐linkage.

## 2 MATERIALS AND METHODS

After Institutional Review Board (IRB) approval, as well as Data Use Approval (DUA), the data was obtained from the National Cancer Institute's (NCI) Surveillance Epidemiology and End Results (SEER) database, 2000–2017.

### 2.1 Study population and Sample

This study involved a tumor registry of a population of children diagnosed with retinoblastoma and followed for the disease. The age group at diagnoses included infants and other children till the age of 19. Both male and female children were included in this population of patients.

### 2.2 Study design

Due to the preexisting nature of the data in the SEER registry, a retrospective (case‐only) cohort study was used to assess the sex variability in CMI, mortality and survival of children with retinoblastoma.

### 2.3 Data source

This study used the most recent and up‐to‐date SEER registry available from 2000 to 2017. The NCI’s SEER data registry was used to obtain CMI, mortality, race, tumor prognostic data, and sociodemographic, as th.^9^ This registry began in 1973 with nine tumor registries and currently has more than 18 registries. The registry first expanded in 1992 to total nine registries, adding five more in the year 2005. The most commonly known registries of the SEER database include 9, 11, 13, 17, and 18. The following information can be obtained from the SEER database, cancer diagnosis, patient demographics, primary tumor site, tumor morphology, stage at diagnosis, prognostic factors, the first course of treatment, and follow‐up for vital status. The SEER registry is the most comprehensive population‐based source of data of cancer stage and survival, with the details of this registry available elsewhere.^10^ Registries for the SEER database must be approved based on the ability of cancer centers to provide high quality, population‐based information, and variables. The classification of cancer is based on the International Classification of Disease 3rd edition (ICD‐O‐3).^11^

### 2.4 Sample size and power estimations

The current study involved preexisting data from the SEER registry, implying a known sample size (*n* = 439). To estimate the statistical power, reflecting the ability of the study to detect the minimum difference of 20% hazard ratio (HR) with respect to survival, should such a difference exist. The following parameters were used for power estimation: (a) sample size comparing the hazard in female unexposed (*n* = 188, event *n* = 2) to hazard in the exposed, males (*n* = 240, event, *n* = 9), (b) type I error tolerance of 1% (99% CI), and (c) Cox Proportional Hazard Model comparing one slope to a reference value. With these parameters, a sufficient power (1 − β) was obtained, 99.9%.

### 2.5 Variables ascertainment

The outcome variable in this study was retinoblastoma mortality that was captured as vital statistics, implying dead and alive. This variable was transformed into a binary scale for utilization in binomial regression as well as Cox proportional hazard model. In the survival analyses utilized in this study, this response variable was characterized as time as the function of dying. This variable was measured on a binary scale, implying that mortality = 1 and alive = 0. The independent variables indicating exposure were race and sex.

### 2.6 Statistical analysis

A pre‐analysis screening was performed to examine data for missing variables and outliers. The categorical or descriptive variables were summarized using frequency and percentages.

### 2.7 CMI rates and trends analysis

A Weighted Least Squares method was used in order to estimate the age‐adjusted retinoblastoma cases in the age groups from 1 to 19 years old. This method allows for the computation of linear and non‐linear regression models.

### 2.8 Signal Amplification and Risk‐Specific Stratification model

The Signal Amplification and Risk‐Specific Stratification (SARSS) modeling technique was used to assess mortality risk by multivariable modeling of social determinants of health. This SARSS model allows for the assessment of the magnitude of confounding prior to adjustment in a multivariable model, magnitude of confounding MAC (=crude‐adjusted/crude × 100). In addition, this model provides the opportunity to assess for the effect measure modification implying clinically and biologically differences in the point estimate by stratum.^12^

To determine the independence of the variables utilized in this study by sex, a chi‐squared statistic and Fischer's exact were used to compensate for the small, expected cell count in the data. The hypothesis‐driven analyses implying the mortality risk modeling by sex was performed using the binomial regression model. This model allows for a risk prediction of the probability of dying given the sex as male as well as female. To determine the risk ratio (RR), the risk in the exposed namely, male was divided by the risk in the unexposed namely, female. This quantification was restricted to univariable modeling. The survival analyses utilized the Cox proportional hazard modeling and met the survival of assumption that the hazard rate remains constant over time. Both the univariable and multivariable Cox proportional model were utilized in assessing the risk of dying as a function of time, given sex of children with retinoblastoma.

Since a single risk factor does not entirely predict the risk, the Cox proportional hazard model at a multivariable level was utilized to control for the confounding. To enter any potentially confounding variable into the model building, such variable was assessed for the MAC. If the MAC was greater than 10% based on MAC, such a variable was entered into the model.^12^

The life table, Kaplan–Meier and Nelson–Helen cumulative hazards were used to illustrate the proportion of survival and cumulative hazard, respectively. Additionally, the log‐rank test was used to examine the quality of survival by sex. Furthermore, the margins plots were used to assess the predictive mean of dying, given the sex of children with retinoblastoma.

All tests were two‐tailed, and the type‐1 error tolerance were set at 5% (95% CI) and 1% (99% CI) for univariable and multivariable models, respectively. The entire analyses were performed using STATA, version 16 (STATA Corporation).

## 3 RESULTS

The current study was proposed to assess the sex differentials in pediatric retinoblastoma survival, as well as examine the CMI, mortality, and survival. This data set utilized information from pediatric patients, diagnosed with this condition in the SEER data between 2000 and 2017. During the study period 2000–2017, there were 439 children diagnosed with retinoblastoma, of these number of cases, 11 experienced mortality. Overall, 2.5% of children with retinoblastoma expired during this study period. The frequency varied by sex and race. Male children (*n* = 249, 56.7%) were more likely to be diagnosed with retinoblastoma compared to female children (*n* = 190, 43.3%). Regarding race, white children (*n* = 344) were more likely to be diagnosed with retinoblastoma compared to their Black/African American counterparts (*n* = 45). The Annual Percent Change (APC) was lower among Black patients (APC = −14.2) relative to White patients (APC = 0.3). A similar observation occurred in sex, where the APC was higher among males (male = 0.4, female = 0.1). The overall percent change (OPC = 16.5) differed by sex (males = 5.1, females = 31.8). There was an increase in percent change during the period of the study, implying increasing incidence with time between 2000 and 2017.

Table 1 illustrates the study characteristics of children diagnosed with retinoblastoma and followed for the disease between 2000 and 2017. There were differences by sex with respect to urbanity, implying where the retinoblastoma patients resided at the time of diagnosis. In addition, household median income did vary by sex but was very marginal, (*χ* ^2^ = 4.69, *df* = 1, *p* = 0.860). Although, not in the table, racial differences were also observed in median income, implying the social determinants of health, *χ* ^2^ = 33.63, *df* = 16, *p* = 0.01. The tumor grade differed by sex, with poorly differentiated malignancy observed more among males relative to females *χ* ^2^ = 108.10, male‐female *p* < 0.001. With respect to tumor primaries, there were more males diagnosed with second primaries compared to females, *χ* ^2^ = 4.08, *df* = 1, *p* = 0.043.

**TABLE 1:** Study characteristics of children with retinoblastoma stratified by sex, Surveillance Epidemiology and End Results data (2000–2017)

	Female		Male		χ^2^ *df*	*p*
	*n*	%	*n*	%		
Age					0.50	0.92
<1 year	54	43.20	71	56.80		
1–4 years	129	43.73	166	56.27		
5–9 years	6	35.29	11	64.71		
10–14 years	1.00	50.00	1	50.00		
Race					0.36	0.99
White	150	43.60	194	56.40		
AI/AN	2	50.00	2	50.00		
Asian/Pacific Islander	17	42.50	23	57.50		
Black	19	42.22	26	57.78		
Unknown	2	33.33	4	66.67		
Grade					0.83	0.84
Well differentiated	57	43.51	74	56.49		
Moderately differentiated	35	46.67	40	53.33		
Poorly differentiated	42	40.00	63	60.00		
Undifferentiated;	56	43.75	72	56.25		
Retinoblastoma history					0.96	0.81
9510/3: Retinoblastoma, NOS	117	43.33	153	56.67		
9511/3: Retinoblastoma, differentiated	21	38.89	33	61.11		
9512/3: Retinoblastoma, undifferentiated	50	45.87	59	54.13		
9513/3: Retinoblastoma, diffuse	2	33.33	4	66.67		
Mortality					2.90	0.09
Alive	188	43.93	240	56.07		
Dead	2	18.18	9	81.82		
Income level					1.68	0.80
<$40,000	13	44.83	16	55.17		
$40,000–49,999	27	47.37	30	52.63		
$50,000–59,999	81	44.02	103	55.98		
$60,000–69,999	31	37.35	52	62.65		
>$70,000	38	44.19	48	55.81		
Urbanity					0.00	0.99
Metropolitan	158	43.29	207	56.71		
Suburban/rural	32	43.24	42	56.76		

Note

The type‐1 error tolerance was set at 5% (0.05). The percentages reflect the row frequency and percentages.

Abbreviations: AI/AN, American Indian/Alaska Native; NOS, not otherwise specified.

**FIGURE 1:**
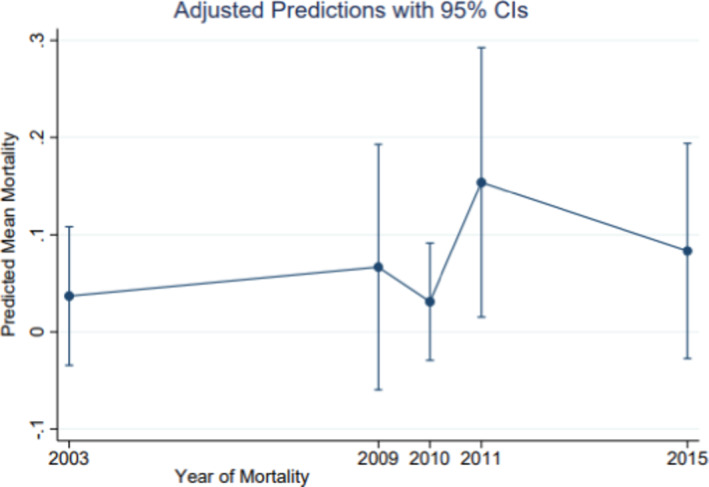
Margins plot of the predictive mean of mortality, 2000–2017. The plot exhibits excess mortality among male children with retinoblastoma as indicated by the upper curve. However, because the confidence intervals implying the lower and the upper bars overlap, there is a precision instability implying cautious optimism in the generalization of this finding beyond this sample

**FIGURE 2:**
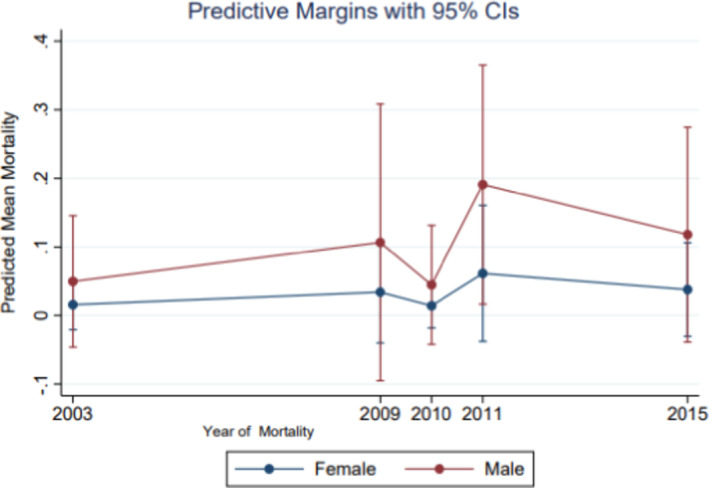
The margins plot of dying from childhood retinoblastoma comparing male and female cases. This predictive margin illustrates the risk of dying which was higher among males compare to females, with the upper line indicative of male mortality

Table 2 demonstrates the relationship between retinoblastoma mortality and sex, implying the assessment of sex as the exposure function of mortality. Additionally, other variables, namely, tumor grade, tumor primaries, urbanity, and household median income, were assessed in regard to mortality. There was a significant sex differential in retinoblastoma mortality. Relative to females, males were three times as likely to die from this malignancy, RR = 3.43, 95% CI [1.0–15.71]. Further, there was a mortality differential with respect to household medium income, whereby there was an inverse dose response observed in childhood retinoblastoma mortality, implying the lower the income the higher the mortality, RR = 0.86, 95% CI [0.08–8.87]. With respect to urbanity and mortality, there was excess mortality in rural compared to metropolitan, RR = 3.28, 95% CI [0.08–8.86]. Compared with well‐differentiated tumors, children with poorly differentiated tumors were 66% more likely to die. Although, imprecise due to the sample size, the RR = 1.55, 95% CI [0.38–7.27]. Although, not shown in the table, compared to localized retinoblastoma, there were increased risk of mortality for other categories. Relative to localized retinoblastoma, children diagnosed with distanced tumor were six times as likely to die, RR = 6.0, 95% CI [1.31–27.41], *p* = 0.02. There was racial variance in mortality between Whites and Asians/Pacific Islanders. Relative to Whites there was a 96% insignificant but clinically meaningful difference in mortality among Asians/Pacific Islanders, RR = 1.96 95% CI [0.41–9.40]. Figure 1 illustrates the predictive margins of dying based on the margins plot stratified by sex of children diagnosed with retinoblastoma and followed for the disease between 2000 and 2017. The upper line represents the mortality among males while the lower line represents the mortality among females, implying excess mortality observed among males relative to females. Additionally, the risk of dying by sex is illustrated in Figure 2. This figure indicates excess mortality among males compared to females based on the effect size and 95% confidence interval, which reflects the precision measures of the point estimate. There is a clear distinction of excess mortality among males relative to females as represented by the margins plot indicating males at the upper curve while females at the lower curve.

**TABLE 2:** Univariable association between sex and mortality in pediatric retinoblastoma, Surveillance Epidemiology and End Results 2000–2017

	Risk ratio	95% CI	*p*
Sex			
Female	1.00	Referent	Referent
Male	3.43	0.75–15.71	0.11
Race			
White	1.0	Referent	Referent
Alaska Indian/Alaska Native^a^	1.00	—	—
Asian/Pacific Islander	1.91	0.43–8.54	0.40
Black^a^	1.00	—	<0.001
Age group			
1–4 years	0.74	0.22–2.49	0.63
4–9 years^a^	1.00	—	—
10–14 years^a^	1.00	—	—
Tumor stage			
Blank(s)	1.07	0.13–8.65	0.95
Distant	6.00	1.31–27.41	0.02
Localized^a^	—	—	—
Regional^a^	1.00	—	—
Un‐staged	6.40	1.41–29.08	0.02
Urbanity			
Metropolitan	1.0	Referent	Referent
Rural/suburban	3.28	0.90–11.93	0.07
Median income			
First quintile	1.00	Referent	Referent
Second quintile	0.86	0.08–8.87	0.90
Third quintile^a^	1.00	—	—
Fourth quintile	0.17	0.02–1.76	0.14
Fifth quintile	0.75	0.10–5.84	0.78

Note

The type‐1 error tolerance was set at 5% (0.05). The median income level was assessed by quintile, namely first quintile (<$40,000), second quintile ($40,000–49,999), third quintile ($50,000–59,999), fourth quintile ($60,000–69,999), and fifth quintile (>$70,000).

^a^ Unable to estimate the risk ratio due to small sample size.

**FIGURE 3:**
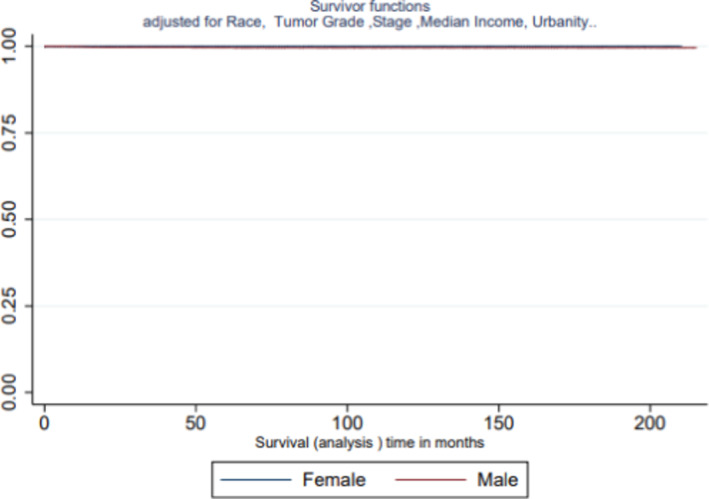
The Kaplan–Meier survival curve of children diagnosed with retinoblastoma between 2000–2017. Overall the mortality is relatively low, indicative of improved survival

**FIGURE 4:**
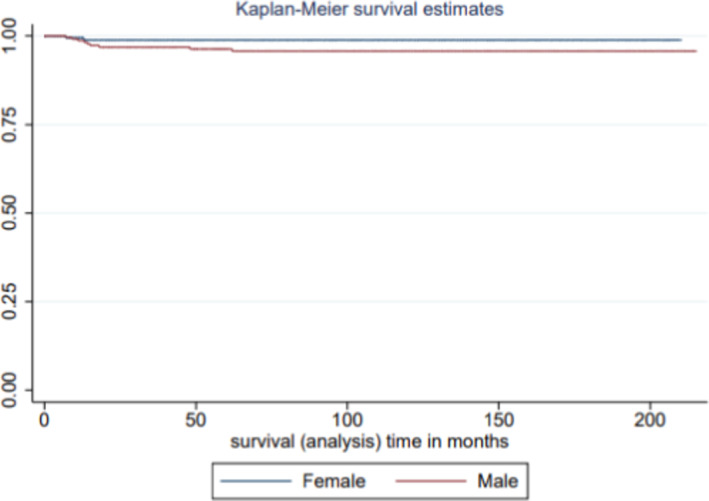
Kaplan–Meier survival curve by sex of children with retinoblastoma, Surveillance Epidemiology and End Results (SEER), 2000–2017. This Kaplan–Meier curve illustrates the survival disadvantage of males with male's survival experience presented in the lower curve

Overall, this sample illustrates a better survival experience compared to retinoblastoma survival variance mapping in other settings. Table 3 exhibits the univariable association between sex as an exposure function of survival and pediatric retinoblastoma. There was a substantial survival disadvantage of males diagnosed with retinoblastoma and followed for the disease between 2000–2017. Compared to female patients, male patients were three times as likely to die from the disease, HR = 3.39, 95% CI [0.73–15.72]. In addition to sex variance in retinoblastoma survival there was survival differences in urbanity and household median income. Regarding household median income, there was a marginal difference in retinoblastoma survival, with survival disadvantage observed among children with lower income. Similarly, survival disadvantage was observed among children residing in rural areas compared to metropolitan. Children diagnosed with this malignancy in rural/suburban areas were more than three times as likely to die from retinoblastoma, HR = 3.43, 95% CI [1.0–12.94]. Since no mortality was observed among American Indian/Alaskan Native and Blacks, there was no risk estimate in these two populations. Figure 3, which is a Kaplan–Meier survival curve, illustrates this experience, where the survival curve is higher without any median survival observed. Figure 4 demonstrates the survival disadvantage of males relative to females based on the Kaplan–Meier Survival Estimate. The upper curve is indicative of the survival advantage of females relative to males.

**TABLE 3:** Retinoblastoma survival by sex and other variables: univariable model of Cox proportional hazard model

	Hazard ratio	95% CI	*p*
Sex			
Female	1.00	Referent	Referent
Male	3.40	1.0–15.72	0.118
Race			
White	1.00	Referent	Referent
Alaska Indian/Alaska Native^a^	—	—	—
Asian/Pacific Islander	2.02	0.44–9.37	0.400
Black^a^	—	—	—
Age group			
>1 year	1.0	Referent	Referent
1–4 years	0.75	0.22–2.57	0.648
5–9 years^a^	—	—	—
10–14 years^a^	—	—	—
15–19	—	—	—
Tumor grade			
Well/moderately differentiated	1.0	Referent	Referent
Poorly differentiated/undifferentiated	1.35	0.40–4.62	0.632
Median income			
<$40,000	1.00	Referent	Referent
$40,000–49,999	0.89	0.08–9.79	0.922
$50,000–59,999^a^	—	—	—
$60,000–69,999	0.14	0.01–1.54	0.108
>$70,000	0.66	0.1–5.47	0.698
Urbanity			
Metropolitan	1.0	Referent	Referent
Rural/suburban	3.43	0.068	0.91–12.95

^a^ Unable to estimate the HR due to small sample size.

**FIGURE 5:**
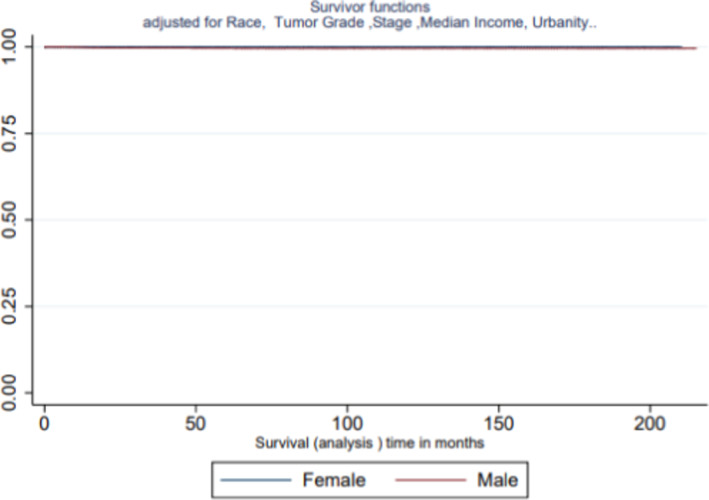
Kaplan–Meier survival curve by sex, adjusted for potentially confounding variables in survival experience of children with retinoblastoma, SEER 2000–017. The Kaplan–Meier adjusted for urbanity, median income, tumor grade, and primaries. While the curve illustrated a slight variance the survival disadvantage of males persisted as indicated in the lower curve

Table 4 illustrates the multilevel association between sex as a function of survival and pediatric retinoblastoma. After controlling for potential confounding variables namely tumor grade, urbanity, and household median income, the survival disadvantage of males persisted. Compared to female children, males were more than three times as likely to die, adjusted HR (aHR) = 3.43, 95% CI [0.37–31.58]. While Asian/Pacific Islander, compared to Whites were more than three times as likely to die, aHR = 3.30, 99% CI [0.37–20.39]. Figure 5 demonstrates the Kaplan–Meier Survival Curve adjusted for race, urbanity, tumor grade, and household median income. Despite this adjustment survival disadvantage of males persisted, with males represented with the lower curve while females were represented with the upper curve.

**TABLE 4:** Multivariable survival by sex of children with retinoblastoma, Surveillance Epidemiology and End Results 2000–2017

Variable	aHR	SE	99% CI
Sex			
Female	1.0	Referent	Referent
Male	3.4	2.95	0.37–31.60
Tumor grade			
Well/moderately differentiated	1.0	Referent	Referent
Poorly differentiated	1.86	1.49	0.24–14.59
Urbanity			
Metropolitan	1.0	Referent	Referent
Rural/suburban	8.55	13.85	0.13–555.1

Note

The multilevel model used in explaining the survival disadvantage of males involved four levels namely: model 1 sex only, model 2 = model1 + age at tumor diagnosis, model 3 = model 2 + tumor prognostic factors (tumor grade, and tumor stage), model 4 = model3 + social determinants of health namely, urbanity. The type 1‐error tolerance was set at 1% (99% CI).

Abbreviation: aHR, adjusted hazard ratio.

^a^ Unable to estimate the HR due to small sample size in the subgroup.

## 4 DISCUSSION

The current study was conceptualized to assess the sex differential in pediatric retinoblastoma CMI, mortality, and survival. The assessment involved the application of the binomial regression model for the mortality variance, while the Cox proportional hazard model was used for the predictors of survival, namely sex as a function of survival. There are a few relevant findings based on this modeling. First, the CMI of pediatric retinoblastoma varied by sex as well as by race. Second, there was excess mortality among males compared to females. Third, male children with retinoblastoma indicated survival disadvantage compared with their female counterparts. Further, after controlling for the potentially confounding variables such as social determinants of health, the sex disparities in retinoblastoma survival persisted, which may be explained by male X‐linkage.

We have demonstrated the increased CMI of retinoblastoma among male children as well as among Blacks and American Indian/Alaska Native.^13^, ^14^ Previous literature have observed increased CMI of several pediatric malignancies among male children compared to female.^2^, ^15^, ^16^ The current study from the SEER registry supports this previous data. However, there remains a limited explanation of the observed variance.^2^ The observed increased CMI of this malignant neoplasm among Whites may be explained by limited access and utilization of cancer treatments and management by Blacks and American Indian/Alaska Native, due to social injustice that reflects lack of insurance as well as limited access to private insurance coverage.^17^ Increased CMI is observed in this study among males; however, there are no clear explanations of this variance. Retinoblastoma is a malignant neoplasm involving a mutation either through inheritance or sporadic.^1^, ^18^ The sex variance in the CMI may be explained by the variability in the function of X‐chromosome among females compared to males. While X‐chromosome is common among males and females, the extra X‐chromosome in females which accounts for an estimated 4% of the total function by estrogen elaboration, may provide some explanation of this variance.^19^ Notwithstanding the role of X‐chromosome in estrogen elaboration associated with 4% of this chromosome, the remaining 96% function of the X‐chromosome among females is not very well understood.^19^ Further the X‐chromosome in females may play a role in programmed cell death (apoptosis), implying its role in the activation of the tumor suppresser gene, p53.^20^

This study has illustrated increased mortality from pediatric retinoblastoma among males relative to females. The observed variance may be explained by the tumor prognostic factor differentials in male and female, such as the tumor grade and primaries. In this current sample, males were observed with, higher proportion of poorly differentiated and undifferentiated retinoblastoma grade, which impacts prognosis and survival.^3^, ^21^ Since tumor grade adversely influences mortality, the observed increased risk of dying among males may be explained by the tumor grade variance, comparing male and female children with retinoblastoma.^22^ Although, not in the table, compared to females, the second primary malignancy characterized as tumor primaries was more common among males. In addition, the mortality variance by sex, where males illustrated higher mortality in this malignancy may also be explained by the variance in tumor primaries implying higher second primary malignancy among males compared to females.^23^

Despite these explanations in accounting for the survival disadvantage of male children relative to their female counterparts, there are still some insufficiencies which requires the potential explanation of this variance based on the male X‐linkage.^16^, ^19^ Retinoblastoma and other malignant neoplasms have been shown with increased risk among male children as well as survival disadvantage in major malignancies, namely acute lymphoblastic leukemia, lymphoma, renal carcinoma, rhabdomyosarcoma, and recently retinoblastoma.^24^ The excess mortality of male children may be explained by the male X‐chromosome with respect to either recessive or dominant allele, which may play a role in cell differentiation, maturation, and proliferation. Therefore, it is highly likely that the X‐linkage in male may be responsible for the persistent male children malignant neoplasm survival disadvantage, but not the female X‐chromosome, implying an urgent need for the evaluation of the male X‐chromosome, such as allosomes in retinoblastoma risk, prognosis, mortality, and survival. In effect this finding implicates urgent assessment of the probability of X‐linkage contribution in apoptosis, pro‐oncogene conversion to oncogene, and tumor suppressor gene, as well as the prognosis with respect to chemotherapy, bone marrow transplant, radiation, and surgical response to cancer therapeutics among male children with retinoblastoma.

We have indicated that males with retinoblastoma experienced survival disadvantage compared to their female counterparts. Survival disadvantaged by sex in some pediatric malignancies have been observed.^10^ These studies clearly observed female children with survival advantage compared to their male counterparts. However, there are no clear explanations of the observed sex variance in survival. With the limited data on the role of X‐chromosome in estrogen elaboration and the potentials in enhancing apoptosis it is plausible that the observed male survival disadvantage may be slightly explained by the protective function of the X‐chromosome with respect to apoptosis enhancement, implying increased program cell death among females compared to males. Also, because of the potentials for tumor suppression based on the X‐chromosome in female there is a possibility of improved prognosis, once diagnosed with this condition and followed for the disease among females, which explains the survival advantage of female compared to male with pediatric retinoblastoma. Further since the tumor grade plays a role in retinoblastoma prognosis as well as survival, the observed survival disadvantage of males may be explained by the higher proportion of males with poorly differentiated and undifferentiated retinoblastoma.^21^ Next, since social determinants of health have been shown to impact on survival in adult malignancies as well as in some pediatric cancers, geographic locale mainly the residence of children at the time of diagnosis, as well as median household income, may explain the sex variance in the pediatric retinoblastoma survival. However, it is highly unlikely that the current data indicated sex differences in the physical environment as well as household median income.

Since a single predictor cannot clearly explain the outcome phenomenon, there remains a need for a plausible explanatory model using a multivariable process in understanding survival of children diagnosed with retinoblastoma and followed for the disease. This study utilized an explanatory model at the multilevel assessment and observed a persistent survival disadvantage of males compared to females. After controlling for tumor prognostic factors such as tumor grade, urbanity, and household median income the survival disadvantage of males persisted, implying limited confoundings in the modeling, indicative of unmeasured confoundings. Previous cancer studies on confounding adjustment have clearly observed the same compared to this study,^17^ in which the point estimate such as adjusted HR remains the same or increased after the adjustment.^25^ The observed median income in this sample, with respect to potentially confounding is supported by the current data on global mapping of retinoblastoma disparities. Specifically, countries with low socioeconomic index compared to those with higher socioeconomic index, such as the United States, are more likely to experience retinoblastoma survival disadvantage among children. The indicated survival disadvantage in this mapping is explained by bilateral retinoblastoma, late‐stage at diagnosis, limited treatment, and management resources, and lack of clinical trials involving immunotherapy, either through infusion or injection in improving the prognosis in treating and inducing remission.^3^, ^8^

We have observed the role of the physical environment characterized as urbanity in retinoblastoma survival. The understanding of the possible role of epigenomic modulation implying gene and environment interaction may explain the survival disadvantage for males in this malignancy.^26^ Epigenomic modulation involves the covalent binding of the methyl group (CH3) with the cytosine‐phosphate‐guanine region of the gene, predisposing to transcription inhibition via the inability of the RNA to translate the transcript (tRNA) into amino acid codon implying impaired protein synthesis.^16^ The outcome of aberrant epigenomic modulation associated with the candidate genes such as RB1 involved in retinoblastoma comparing male and female may provide additional explanation with respect to the survival disadvantage of males in this condition. Epigenomic studies stratified by sex with respect to DNA methylation allows for the specific risk characterization and induction therapy prior to the standard of care namely chemotherapy, radiation, monoclonal antibodies, surgery, and bone marrow transplants.

Despite the strengths of this study namely large sample size and adequate explanatory model, there are some limitations. First, this study involved the assessment of a preexisting data using a retrospective cohort design which has the potentials for selection, information, and misclassification biases. However, it is highly unlikely that the findings of male survival disadvantage are influenced solely by these biases. Second, these findings may be driven by unmeasured confoundings implying the recommendation of data on the treatment received, health insurance, and social determinants of health to be provided by the NIH, NCI, and SEER. Therefore, such recommendation if provided will result in a full understanding of the factors associated with survival differences in order to carefully map intervention in narrowing and ultimately eliminating the sex disparities in pediatric retinoblastoma.

## 5 CONCLUSION

In summary, pediatric retinoblastoma survival varied by sex, with males presented with the survival disadvantage compared to females. These findings are suggestive of the need to conduct epigenomic studies by sex in order to initiate induction therapy, avoid chemotherapy dose escalation, toxicity, and improve survival of children diagnosed with retinoblastoma and followed for the disease. Based on these findings, we recommend policy formulation, implementation, and evaluation in assessing the social determinants of health as well as social injustice for health disparities narrowing and ultimate elimination in pediatric retinoblastoma survival as well as overall pediatric malignancies.

## CONFLICT OF INTEREST

All authors declared no conflict of interest.

## AUTHOR CONTRIBUTION

LH conceptualized the study, directed the analysis and interpretation, drafted the manuscript, reviewed and approved the final draft. JB directed the data processing, facilitated the draft of the manuscript, interpreted the output, reviewed and approved the final draft. EP assisted in data processing, reviewed the studies significance, assisted in manuscript draft, reviewed and approved the draft. BB assisted in the manuscript draft, reviewed and approve the draft. DH assisted in the manuscript draft reviewed and approved the draft. NB, assisted in the manuscript draft, reviewed and approved the draft. KP assisted in the manuscript draft, reviewed and approved the draft. LP assisted in the manuscript draft, reviewed and approve the draft.

## ETHICS STATEMENT

This study received Institutional Review Board (IRB) approval, as well as Data Use Approval (DUA), in order to obtain the data source. SEER is not responsible for the design, analysis, interpretation of the data. This remains the sole responsibility of the investigative team and primary author LH.

## Data Availability

This study used the most recent and up to date SEER registry available from 2000 to 2017. The NCI’s SEER data registry was used to obtain CMI, morality, race, tumor prognostic data, sociodemographic, as well as the social determinants of health.^8^
